# Supplementary material for: Safety and efficacy of 3- and 5-day regimens of levamisole in loiasis: a randomized, placebo-controlled, double-blind clinical trial
Source: Nat Commun. 2025 Jul 4;16:6191. doi: 10.1038/s41467-025-61479-6 (PMC12227723; doi:10.1038/s41467-025-61479-6)
Supplement: Supplementary file 1 — Supplementary Information [file 41467_2025_61479_MOESM1_ESM.pdf]

Safety and efficacy of 3 and 5 days regimens levamisole in loiasis: a randomized, placebo-controlled, double-blind clinical trial – Supplementary Information.

Adverse events	PLA (N=30)	LEV-3 (N=33)	LEV-5 (N=29)
**Gastrointestinal disorders**	7 (23.3)	5 (15.2)	2 (6.9)
Abdominal pain	0	1	0
Colopathy	1	0	0
Diarrhea	2	0	0
Epigastric pain	3	0	1
Meteorism	0	1	0
Nausea	1	3	1
**Eye disorders**	1 (0,3)	0	3 (10.3)
Conjunctival hyperaemia	1	0	2
Xeropthalmia	0	0	1
**Skin disorders**	7 (23.3)	4 (12.1)	2 (6.9)
Cutaneous cysts	0	0	1
Folliculitis	1	0	0
Pruritus	6	3	1
Urticaria	0	1	0
**General disorders**	3 (10.0)	10 (27.5)	5 (17.2)
Asthenia	2	6	0
Fever	0	1	4
Flu-like syndrome	1	3	1
**Nervous system disorders**	3 (10.0)	8 (24.2)	5 (17.2)
Insomnia	0	1	0
Headache	2	6	2
Paresthesia	1	0	1
Vertigo	0	1	2
**Musculoskeletal disorders**	2 (6.7)	2 (6.1)	0
Arthralgia	0	1	0
Myalgia	2	1	0

Table 1. Classification of the adverse events (AEs) possibly related to treatment, and of the subjects having developed such AEs in the three treatment arms.
Data presents the total number of AEs by symptom type, with percentages representing the proportion of individuals experiencing each symptom in each group. For the summary by organ systems (gastrointestinal, eye, skin, general disorders, and musculoskeletal disorders), percentages represent the proportion of individuals who reported symptoms within each category among the total number of individuals included in each group.

	Adjusted Odds-ratio	95% Confidence Interval	P value
Age (in continuous)	1.01	0.97-1.05	0.706
Sex			
Female	Ref.		
Male	1.81	0.67-4.90	0.240
Loa loa MFD (mf/mL) at D1			
< 20,000	Ref.		
20,000-29,999	0.85	0.22-3.33	0.821
≥ 30,000	2.10	0.52-8.55	0.299
Arms			
PLA	Ref.		
LEV-3	1.14	0.33-3.88	0.840
LEV-5	0.22	0.05-0.85	0.029
Loa loa MFD reduction rate at D3			
< 25%	Ref.		
25-49%	3.94	1.02-15.26	0.047
≥ 50%	3.19	0.78-13.19	0.108

Table 2. Logistic regression of the risk of adverse events occurred during the follow-up.
Logistic regression model with two-sided Wald tests. No adjustment for multiple comparisons was applied. Exact p-values are reported where applicable. 95% CI: 95% confidence interval.

	Adjusted beta-coefficients [95% CI]	P-value
Ref. Day 1 (for Arm PLA)		
Day 3	1.28 [-8.64; 11.21]	0.800
Day 5	-0.03 [-10.06; 9.99]	0.995
Day 7	0.03 [-10.73; 10.80]	0.995
Day 15	7.94 [-2.93; 18.80]	0.152
Day 30	7.36 [-6.09; 20.81]	0.284
Ref. Arm: PLA (at Day 1)		
Arm: LEV 3 mg/kg	0.50 [-13.61; 14.62]	0.945
Arm: LEV 5 mg/kg	-0.42 [-15.14; 14.31]	0.956
Interactions between days and arms		
Day 3 × LEV 3	-31.25 [-45.07; -17.43]	< 0.001
Day 3 × LEV 5	-31.46 [-45.81; -17.11]	< 0.001
Day 5 × LEV 3	-27.38 [-41.26; -13.51]	< 0.001
Day 5 × LEV 5	-41.41 [-55.89; -26.94]	< 0.001
Day 7 × LEV 3	-17.36 [-32.33; -2.38]	0.023
Day 7 × LEV 5	-40.26 [-56.44; -24.08]	< 0.001
Day 15 × LEV 3	-13.35 [-28.41; 1.71]	0.082
Day 15 × LEV 5	-22.91 [-38.64; -7.18]	0.004
Day 30 × LEV 3	-7.62 [-26.22; 10.98]	0.422
Day 30 × LEV 5	-22.61 [-42.00; -3.22]	0.022
Age (in continous, years)	-0.28 [-0.67; 0.11]	0.164
Sex: Male	3.31 [-6.88; 13.49]	0.525
Baseline *Loa* MFD (Ref. < 20,000 mf/mL)		
20,000 - 30,000 mf/mL	22.58 [14.98; 30.17]	< 0.001
> 30,000 mf/mL	42.70 [33.49; 51.91]	< 0.001
Random-effects parameters		
Variance of slope (Timepoint)	0.77 [0.44; 1.34]	
Variance of intercept	402.40 [256.12; 632.23]	
Covariance slope–intercept	9.56 [3.65; 15.47]	
Residual variance	382.58 [328.15; 446.02]	

Table 3. Mixed linear regression model over time on the *Loa loa* microfilarial densities pre-treatment percentage.

Pairwise comparisons using two-sided Wald tests. Exact p-values are reported. No correction for multiple testing was applied. 95% CI: 95% confidence interval.

	Arms			*P*-values			
**Median and Interquartile Ranges (IQR) of *Loa loa* MFD (mf/mL)**							
Day	PLA (N=24)	LEV-3 (N=26)	LEV-5 (N=18)	a	b	c	d
D1	16,332 (8,170-27,522)	13,120 (8,025-24,675)	16,277 (5,650-27,580)	0.687	0.599	0.561	0.426
D3	14,660 (8,780-35,867)	7,552 (3,795-12,990)	7,347 (6,630-14,770)	0.009	0.005	0.026	0.335
D5	18,612 (7,990-26,115)	9,770 (4,640-13,670)	5,780 (2,165-11,730)	0.004	0.012	0.003	0.207
D7	15,187 (9,567-30,832)	9,940 (3,855-12,645)	5,902 (1,985-17,520)	0.009	0.021	0.006	0.207
D15	12,507 (7,692-33,432)	10,565 (4,640-13,670)	7,750 (2,570-17,530)	0.112	0.112	0.077	0.302
D30	13,842 (7,807-38,075)	13,250 (7,515-20,555)	7,812 (3,485-16,885)	0.118	0.141	0.059	0.268
**Median and IQR of the *Loa loa* MFD reduction compared to the pre-treatment result (D1)**							
Day	PLA (N=30)	LEV-3 (N=33)	LEV-5 (N=28)	a	b	c	d
D3	-3.5 (-11.0-17.0)	38.4 (19.1-51.8)	25.7 (21.1-46.5)	< 0.001	< 0.001	0.002	0.285
D5	-1.6 (-8.7-14.0)	31.5 (14.2-58.1)	51.0 (17.9-63.6)	< 0.001	< 0.001	< 0.001	0.129
D7	8.2 (-13.2-19.0)	24.3 (12.1-51.0)	34.4 (25.5-62.9)	< 0.001	0.004	< 0.001	0.035
D15	-1.4 (-25.9-16.4)	26.0 (-16.9-40.8)	16.4 (5.1-40.8)	0.087	0.099	0.057	0.277
D30	-2.7 (-39.2-28.6)	17.6 (-26.3-37.7)	28.5 (-13.7-54.5)	0.124	0.141	0.062	0.279

Table 4. Median microfilarial density (MFD), and median relative difference in MFD between DX (X = 3, 5, 7, 15, or 30) and D1, by arm for per-protocol analyses

Two-sided Kruskal-Wallis non parametric test: a) between the three arms-and *post hoc* analyses with Dunn's pairwise test using a Holm correction for multiple tests: b) between LEV-3 and PLA arms, c) between LEV-5 and PLA arms, and d) between LEV-5 and LEV-3 arms. Exact P-value are reported.

**Proportion of individuals with MFD reduction >40%**								
		PLA	LEV-3	LEV-5	a	b	c	d
**D3**	No	22 (91.7)	13 (50.0)	12 (66.7)	0.012	0.004	0.100	0.216
	Yes	2 (8.3)	13 (50.0)	6 (33.3)				
**D5**	No	21 (84.5)	15 (57.7)	6 (33.3)	0.003	0.040	< 0.001	0.099
	Yes	3 (12.5)	11 (42.3)	12 (66.7)				
**D7**	No	22 (91.7)	17 (65.4)	10 (55.6)	0.051	0.054	0.036	0.364
	Yes	2 (8.3)	9 (34.6)	8 (44.4)				
**D15**	No	21 (84.5)	19 (73.1)	13 (72.2)	0.716	0.716	0.716	0.716
	Yes	3 (12.5)	7 (26.9)	5 (27.8)				
**D30**	No	22 (91.7)	20 (76.9)	9 (50.0)	0.033	0.151	0.012	0.126
	Yes	2 (8.3)	6 (23.1)	9 (50.0)				
**Proportion of individuals with MFD reduction >80%**								
		PLA	LEV-3	LEV-5	a	b	c	d
**D3**	No	24 (100.0)	23 (83.5)	17 (94.4)	0.717	0.532	0.858	0.858
	Yes	0	3 (11.5)	1 (5.6)				
**D5**	No	24 (100.0)	24 (92.3)	17 (94.4)	0.878	0.995	0.778	0.854
	Yes	0	2 (7.7)	1 (5.6)				
**D7**	No	24 (10.0)	24 (92.3)	15 (83.3)	0.284	0.530	0.284	0.530
	Yes	0	2 (7.7)	3 (16.7)				
**D15**	No	23 (95.8)	24 (92.3)	17 (94.4)	0.999	0.899	0.978	0.988
	Yes	1 (4.2)	1 (7.7)	1 (5.6)				
**D30**	No	23 (95.8)	26 (100.0)	16 (88.9)	0.648	0.782	0.782	0.648
	Yes	1 (4.2)	0	2 (11.1)				

Table 5. Proportion of participants with a 40% and 80% reduction in their microfilarial density (MFD) per arm for per-protocol analyses

Two-sided Fisher's exact test with Holm correction for multiple test (adjusted-*P* values): a) between the three arms, b) between LEV-3 and PLA arms, c) between LEV-5 and PLA arms, and d) between LEV-5 and LEV-3 arms. Exact P-value are reported.

Text 1. Classification of adverse events.

Any reaction occurring in a person who is a subject of research involving the human subject whether or not the event is related to the research or to the administration of the drug is defined as an adverse event (AE). Any harmful and undesired reaction following administration of the drug, or any incident that could have resulted in such a reaction if appropriate action had not been taken, in an individual who is a research subject is defined as an adverse drug reaction (ADR). Any AE or ADR meets the definition of "serious" if it results in death, endangers the life of the participant, requires hospitalization or prolonged hospitalization-causes significant or lasting disability or incapacity-results in a congenital anomaly or malformation or is considered by investigators as a significant medical event. The intensity of all AEs (serious and non-serious) has been assessed according to the ICH guidelines: mild, moderate, severe or life-threatening [1].

The intensity of all clinical AEs (severe and non-serious) has been assessed according to the following list:

- Grade 1 Mild or transient discomfort, without limitation of usual daily activity-does not require medical intervention or corrective treatment.
- Grade 2 Moderate Partial limitation of usual daily activity-medical intervention or corrective medical intervention or corrective treatment may not be necessary.
- Grade 3 Severe Limitation of usual daily activity-requires medical intervention and corrective treatment, hospital admission possible.
- Grade 4 Life-threatening Very limited activity-requires medical intervention and corrective treatment, almost always in hospital.

However, biological AEs found on further investigation in the event of an SAE will be assessed according to the CTCAE grading scale version 5.0 and reported by the investigator in the case report form.

Text 2. Protocol summary

"Randomized, double-blind trial evaluating the tolerability and efficacy of 3 and 5 days of levamisole 2.5 mg/kg treatment in *Loa loa* microfilaremic patients."

**Scientific background to the project**	Onchocerciasis and loiasis are vector-borne parasitic diseases caused by Onchocerca volvulus (Ov) and Loa loa (Loa), respectively. Onchocerciasis is endemic in 31 African countries, as well as in a large area along the Venezuela–Brazil border and in Yemen, while loiasis is restricted to Central Africa. It is estimated that approximately 37 million people are infected with Ov and 15 million with Loa.<br><br>Since the early 1990s, endemic African countries have implemented onchocerciasis control programs based on annual mass drug administration (MDA) with ivermectin (IVM). In hyper- and mesoendemic areas, where the disease is a significant public health concern, all individuals aged 5 years and older are offered treatment without prior diagnosis. In 2010, the African Programme for Onchocerciasis Control (WHO/APOC) shifted its goal from disease control to elimination of infection, requiring interventions to be extended to hypoendemic areas. This objective was maintained by the ESPEN initiative, launched in 2016 following the closure of APOC. According to the WHO's recent roadmap for neglected tropical diseases (NTDs), the number of countries with verified interruption of onchocerciasis transmission is expected to increase from 4 in 2020 to 12 by 2030.<br><br>Loiasis is not currently targeted by any specific control program, but it poses a major obstacle to onchocerciasis elimination efforts in co-endemic areas, as IVM can cause severe encephalopathies in individuals with high Loa loa microfilarial densities (MFD > 30,000 mf/mL). These serious adverse events (SAEs) are due to the embolization of large numbers of paralyzed microfilariae in cerebral capillaries. Since 1990, over 500 cases of SAEs (including 60 deaths) have been reported following IVM MDA for onchocerciasis. In hypoendemic co-endemic areas, the risk of post-IVM SAEs outweighs the individual benefits of treatment.<br><br>To date, there is no short-term prospect of a treatment that is both effective against onchocerciasis and safe for individuals with high Loa MFD. Moreover, treating individuals with high Loa MFD remains a challenge due to the risk of SAEs. Several alternative strategies (AS) have been proposed for treating hypoendemic onchocerciasis areas co-endemic with loiasis. One such strategy involves pre-treatment of the population with a safe drug that gradually reduces Loa MFD below the SAE risk threshold, followed by safe administration of IVM to the entire population. Various drugs have been tested in this context, but none have proven satisfactory due to either excessive or insufficient efficacy, or high inter-individual variability in response.<br><br>Levamisole (LEV) is a widely used anthelmintic in Africa (marketed as Decaris®), primarily for soil-transmitted helminths. It has also shown moderate efficacy against O. volvulus and Wuchereria bancrofti (the causative agent of lymphatic filariasis). In 2021, we conducted the first randomized, placebo-controlled clinical trial to evaluate the safety of single-dose LEV in individuals infected with Loa loa, and its effect on microfilarial density. We demonstrated that this regimen was safe even in individuals with high MFDs (up to 70,000 mf/mL). The efficacy of a single 2.5 mg/kg dose of LEV was modest—sufficient to support a gradual reduction strategy, but not enough to significantly lower initial MFD.<br><br>These encouraging results support the rationale for the present project, which aims to evaluate longer LEV regimens (3 and 5 days) for the same indication.

**Description of the study and sample collection**	The objective of this project is to evaluate the safety and efficacy of a 3-day and 5-day regimen of levamisole (LEV) at 2.5 mg/kg for the management of Loa loa microfilaremia. This will be a phase IIb randomized, double-blind, three-arm clinical trial comparing the following groups:<br>- LEV 2.5 mg/kg for 3 days followed by 2 days of placebo,<br>- LEV 2.5 mg/kg for 5 days,<br>- Placebo for 5 days.<br>The only biological samples collected will be standardized thick blood smears (TBS) to measure Loa loa microfilarial density. A total of seven visits are planned for TBS collection throughout the study period. Each TBS requires only 70 µL of capillary blood, obtained via a simple finger prick.
**Ethical considerations**	The study will be conducted in compliance with local regulations. Full information about the study objectives and procedures will be provided orally by the investigators. Additional information will also be shared with local authorities (CDDS, Health Area Chiefs, village leaders). During these sessions, the population will have the opportunity to ask questions before deciding whether to participate in the study.<br>All participants will receive an information sheet, and written informed consent will be required. Participation is voluntary, and individuals may withdraw from the study at any time. It will also be explained that all data will be anonymized, but participants may request access to their individual results or to the overall study findings at any time.<br>The study will cover any adverse events (AEs) that may occur during the trial, and insurance coverage will be provided.<br>To date, there are no specific precautions regarding the use of levamisole in individuals infected with Loa loa microfilariae. However, some risks may exist: Mmild to moderate adverse events, a theoretical risk of grade 3 adverse events. In such cases, participants will be informed that the study team will provide full medical care.<br>These risks will be clearly explained in the participant information and consent form. Serious adverse events are particularly associated with prolonged use of levamisole and specific conditions (e.g., inflammatory diseases, cancer treatments, or concomitant cocaine use). Outside of these contexts, serious adverse events are considered very rare.<br>Regarding biological sampling, only a finger-prick will be required at each visit. Disinfection will be performed according to medical standards prior to each blood collection.
**Relevance of expected results**	We expect that the doses of LEV to be administered (2.5 mg/kg) will not result in any serious adverse events (SAEs). We anticipate a gradual reduction in Loa loa microfilarial density (MFD), but at a faster rate than that observed following a single 2.5 mg/kg dose of LEV.<br><br>If this is not the case, our findings will allow us to definitively rule out the alternative strategy of LEV pre-treatment for the management of high Loa loa microfilaremia. However, repeated LEV administration may still remain a therapeutic option for individual case management in patients with high MFD.<br><br>We also hope that the reduction in Loa loa MFD will be relatively consistent across individuals, allowing for the implementation of mass pre-treatment with LEV as a safe approach to enable Test-and-Not-Treat (TIDC) strategies in co-endemic areas, without the need to rely on Test-and-

	Treat (TNT) strategies.
**Relevant bibliographical references related to the project**	Gardon J, Gardon-Wendel N, Demanga-Ngangue P, Kamgno J, Chippaux J, Boussinesq M. Serious reactions after mass treatment of onchocerciasis with ivermectin in an area endemic for *Loa loa* infection. The Lancet. 1997;350: 18–22.<br><br>Kamgno J, Pion SD, Chesnais CB, Bakalar MH, D’Ambrosio M V., Mackenzie CD, et al. A Test-and-Not-Treat Strategy for Onchocerciasis in *Loa loa* –Endemic Areas. New England Journal of Medicine. 2017;377: 2044–2052. doi:10.1056/nejmoa1705026<br><br>Campillo J, Bikita P, Hemilembolo M, Louya F, Missamou F, Pion S, et al. Safety and efficacy of levamisole in loiasis: a randomized, placebo-controlled, double-blind clinical trial. Clin Infect Dis. 2022;75: 19–27.
