# Supplementary material for: Safety and efficacy of 3- and 5-day regimens of levamisole in loiasis: a randomized, placebo-controlled, double-blind clinical trial
Source: Nat Commun. 2025 Jul 4;16:6191. doi: 10.1038/s41467-025-61479-6 (PMC12227723; doi:10.1038/s41467-025-61479-6)
Supplement: Supplementary file 3 — Reporting Summary [file 41467_2025_61479_MOESM3_ESM.pdf]

nature portfolio

Corresponding author(s): Jérémy T. Campillo

Last updated by author(s): May 16, 2025

Reporting Summary

Nature Portfolio wishes to improve the reproducibility of the work that we publish. This form provides structure for consistency and transparency in reporting. For further information on Nature Portfolio policies, see our Editorial Policies and the Editorial Policy Checklist.

Statistics

For all statistical analyses, confirm that the following items are present in the figure legend, table legend, main text, or Methods section.

n/a	Confirmed	
☐	☒	The exact sample size (n) for each experimental group/condition, given as a discrete number and unit of measurement
☐	☒	A statement on whether measurements were taken from distinct samples or whether the same sample was measured repeatedly
☐	☒	The statistical test(s) used AND whether they are one- or two-sided<br>*Only common tests should be described solely by name; describe more complex techniques in the Methods section.*
☐	☒	A description of all covariates tested
☐	☒	A description of any assumptions or corrections, such as tests of normality and adjustment for multiple comparisons
☐	☒	A full description of the statistical parameters including central tendency (e.g. means) or other basic estimates (e.g. regression coefficient) AND variation (e.g. standard deviation) or associated estimates of uncertainty (e.g. confidence intervals)
☐	☒	For null hypothesis testing, the test statistic (e.g. F, t, r) with confidence intervals, effect sizes, degrees of freedom and P value noted<br>*Give P values as exact values whenever suitable.*
☒	☐	For Bayesian analysis, information on the choice of priors and Markov chain Monte Carlo settings
☒	☐	For hierarchical and complex designs, identification of the appropriate level for tests and full reporting of outcomes
☒	☐	Estimates of effect sizes (e.g. Cohen's d, Pearson's r), indicating how they were calculated

Our web collection on statistics for biologists contains articles on many of the points above.

Software and code

Policy information about availability of computer code

Data collection	We used RedCap, a secure web application for creating and managing online surveys and databases, provided by our organization (IRD).
Data analysis	The data were extracted from RedCap after being double-checked and imported into STATA version 18. All statistical analyses were performed using Stata 18 (StatCorps LP, College Station, Texas, USA). The figures were created using R software (version 4.3.2)

For manuscripts utilizing custom algorithms or software that are central to the research but not yet described in published literature, software must be made available to editors and reviewers. We strongly encourage code deposition in a community repository (e.g. GitHub). See the Nature Portfolio guidelines for submitting code & software for further information.

Data

Policy information about availability of data

All manuscripts must include a data availability statement. This statement should provide the following information, where applicable:

- Accession codes, unique identifiers, or web links for publicly available datasets
- A description of any restrictions on data availability
- For clinical datasets or third party data, please ensure that the statement adheres to our policy

The data that support the findings of this study are openly available in DataSuds repository (IRD, France). Data reuse is granted under CC-BY license.

Research involving human participants, their data, or biological material

Policy information about studies with human participants or human data. See also policy information about sex, gender (identity/presentation), and sexual orientation and race, ethnicity and racism.

Reporting on sex and gender	Findings apply to both sexes. Sex was assigned but not considered in the study design. Due to the limited sample size, sex or gender-based analyses were not conducted.
Reporting on race, ethnicity, or other socially relevant groupings	n/a
Population characteristics	Our study population consisted of 92 adults identified during a screening for loiasis. The mean age was 47.7 years, and women represented 33% of the participants. The mean microfilarial density at baseline was 17,348 mf/mL. The population included in the study lives in a rural area of the Lékoumou Department in the Republic of the Congo. Participants were ultimately assigned to one of three treatment groups: a placebo group, a group receiving levamisole at a dose of 2.5 mg/kg for three consecutive days, and a group receiving levamisole for five consecutive days.
Recruitment	A total of 2,808 individuals were screened in July 2023, of whom 152 met the eligibility criteria for the pre-inclusion phase in June 2024 (Fig. 1). At the inclusion phase, 39 individuals had relocated during the interim period, 2 declined to participate, and 14 were excluded based on non-inclusion criteria. Ultimately, a total of 97 individuals were randomly assigned to one of the three intervention arms.
Ethics oversight	This study was approved by the Committee on Ethics of the Foundation for Medical Research in Congo (N° 51/CEI/FCRM/2024). An Administrative Authorization (N° 000056/MSP/DGSSSa/DPM-19) was released by the Ministry of Health and Population of the Republic of the Congo. The French National Commission on Informatics and Liberty (CNIL DR-2024-099) approved that the study protocol was ensuring compliance with data protection regulations.

Note that full information on the approval of the study protocol must also be provided in the manuscript.

Field-specific reporting

Please select the one below that is the best fit for your research. If you are not sure, read the appropriate sections before making your selection.

☒ Life sciences ☐ Behavioural & social sciences ☐ Ecological, evolutionary & environmental sciences

For a reference copy of the document with all sections, see nature.com/documents/nr-reporting-summary-flat.pdf

Life sciences study design

All studies must disclose on these points even when the disclosure is negative.

Sample size	Sample size calculation<br>Although our primary objective was safety, the absence of any data on SAE risk from our previous clinical trial, and the fact that SAE risk in loiasis depends directly on the number of microfilariae paralyzed and/or destroyed in the first 24–48 hours post-treatment, led us to base our sample-size calculation on treatment efficacy (from the observed in our prior trial [13]). In that trial, 17.4% of participants who received a single 2.5 mg/kg dose of LEV showed a ≥40% reduction in their initial MFD two days after treatment, compared to 1.2% in the placebo group. Assuming an additive effect over a longer treatment period (with a 3-day treatment expected to be three times more effective than a single dose), we estimated that a 3-day treatment would reduce initial MFD by ≥40% in 52.2% of subjects, and a 5-day treatment would achieve this in 87.0% of subjects. Based on these estimates, we calculated that including 27 participants per treatment group would provide 80% power to detect these differences in MFD reduction. To account for a 20% loss to follow-up, we aimed to enrol a total of 99 subjects (33 per group).
Data exclusions	Technical issues related to the slide staining process occurred for 23 individuals at the D7 time point. As the MFD values for this group were deemed unreliable, these individuals were excluded from the per protocol (PP) analyses, leaving the following numbers in the three arms: PLA=24, LEV-3=26, and LEV-5=18 (Total: 68).
Replication	We performed a PP and a mITT analysis
Randomization	A 1:1:1 randomization of 3 arms (LEV-3, LEV-5 or PLA). with blocks size of 3 was performed. Randomization was carried out by an independent statistician, with stratification based on sex, age and Loa MFD.
Blinding	For the study, sealed containers were prepared, each containing the appropriate number of LEV tablets —based on the participant's weight— or matching placebo. Throughout the five-day treatment period, participants took their medication under the supervision of the trial's physicians. Individuals randomized to the LEV-5 arm received 5 days of LEV, those in the LEV-3 arm received LEV from D1 to D3 then placebo from D4 to D5, and those in the PLA arm received placebo for 5 days. All procedures were double-blinded.

Reporting for specific materials, systems and methods

We require information from authors about some types of materials, experimental systems and methods used in many studies. Here, indicate whether each material, system or method listed is relevant to your study. If you are not sure if a list item applies to your research, read the appropriate section before selecting a response.

Materials & experimental systems

n/a	Involved in the study
☒	☐ Antibodies
☒	☐ Eukaryotic cell lines
☒	☐ Palaeontology and archaeology
☒	☐ Animals and other organisms
☐	☒ Clinical data
☒	☐ Dual use research of concern
☒	☐ Plants

Methods

n/a	Involved in the study
☒	☐ ChIP-seq
☒	☐ Flow cytometry
☒	☐ MRI-based neuroimaging

Clinical data

Policy information about clinical studies

All manuscripts should comply with the ICMJE guidelines for publication of clinical research and a completed CONSORT checklist must be included with all submissions.

Clinical trial registration	NCT06252961
Study protocol	Study protocol is available in DataSuds repository (IRD, France)
Data collection	Data were collected during June 2024 in the RedCap software.
Outcomes	The primary objective of the trial was to evaluate the safety of multiple-dose LEV in individuals with Loa microfilaremia. The primary outcome measures were (i) the occurrence of an SAE and (ii) the frequency of AEs during the first month post-treatment.<br>The secondary objective was to assess the effect of LEV on L. loa MFD measured by: (i) the MFD reduction rates at D3, D5, D7, D15, and D30, (ii) the proportions of subjects with MFD reduction rates ≥40% and/or ≥80% at each time point post-D1, and (iii) the efficacy comparison between our arms. Reduction rates (%) were calculated as follows: ((MFD at D1) - (MFD at DX))/(MFD at D1) multiplied by 100 with X=3, 5, 7, 15 or 30 days. Finally, we evaluated the proportion of individuals whose MFD fell below the thresholds of 30,000 mf/mL at D5 and D7.

Plants

Seed stocks	*Report on the source of all seed stocks or other plant material used. If applicable, state the seed stock centre and catalogue number. If plant specimens were collected from the field, describe the collection location, date and sampling procedures.*
Novel plant genotypes	*Describe the methods by which all novel plant genotypes were produced. This includes those generated by transgenic approaches, gene editing, chemical/radiation-based mutagenesis and hybridization. For transgenic lines, describe the transformation method, the number of independent lines analyzed and the generation upon which experiments were performed. For gene-edited lines, describe the editor used, the endogenous sequence targeted for editing, the targeting guide RNA sequence (if applicable) and how the editor was applied.*
Authentication	*Describe any authentication procedures for each seed stock used or novel genotype generated. Describe any experiments used to assess the effect of a mutation and, where applicable, how potential secondary effects (e.g. second site T-DNA insertions, mosiacism, off-target gene editing) were examined.*
